# Deep generative modeling of the human proteome reveals over a hundred novel genes involved in rare genetic disorders

**DOI:** 10.21203/rs.3.rs-3740259/v1

**Published:** 2024-01-04

**Authors:** Rose Orenbuch, Aaron W. Kollasch, Hansen D. Spinner, Courtney A. Shearer, Thomas A. Hopf, Dinko Franceschi, Mafalda Dias, Jonathan Frazer, Debora S. Marks

**Affiliations:** 1 Marks Group, Department of Systems Biology, Harvard Medical School, Boston, MA, USA.; 2 Scientific Consulting, 85435 Erding, Germany.; 3 Dias & Frazer Group, Centre for Genomic Regulation (CRG),The Barcelona Institute of Science and Technology, Barcelona, Spain.; 4 University Pompeu Fabra, Barcelona, Spain.; 5 Broad Institute of MIT and Harvard, Cambridge, MA, USA.

## Abstract

Identifying causal mutations accelerates genetic disease diagnosis, and therapeutic development. Missense variants present a bottleneck in genetic diagnoses as their effects are less straightforward than truncations or nonsense mutations. While computational prediction methods are increasingly successful at prediction for variants in *known* disease genes, they do not generalize well to other genes as the scores are not calibrated across the proteome^1–6^. To address this, we developed a deep generative model, popEVE, that combines evolutionary information with population sequence data^7^ and achieves state-of-the-art performance at ranking variants by severity to distinguish patients with severe developmental disorders^8^ from potentially healthy individuals^9^. popEVE identifies 442 genes in patients this developmental disorder cohort, including evidence of 123 novel genetic disorders, many without the need for gene-level enrichment and without overestimating the prevalence of pathogenic variants in the population. A majority of these variants are close to interacting partners in 3D complexes. Preliminary analyses on child exomes indicate that popEVE can identify candidate variants without the need for inheritance labels. By placing variants on a unified scale, our model offers a comprehensive perspective on the distribution of fitness effects across the entire proteome and the broader human population. popEVE provides compelling evidence for genetic diagnoses even in exceptionally rare single-patient disorders where conventional techniques relying on repeated observations may not be applicable.

## Introduction

Even if every human were sequenced and their phenotypes recorded, the space of disease-causing genetic variation is too large to be studied by population variation alone. Patients with unique sets of symptoms and genotypes would still go undiagnosed. The biodiversity of life on earth provides a deeper view of genetic variation across billions of years of evolution, providing a unique opportunity to uncover complex genetic patterns preserved to maintain fitness. Thus, models that can distill such information have the potential to massively accelerate our ability to leverage genetics for diagnosis, preventive care, and treatment.

In the context of severe genetic disorders likely to be caused by a single variant, the task is to identify the causal variant amongst the millions of mutations in a single individual. One powerful approach is the sequencing of trios – patient and their parents – which provides a way to narrow down the candidate pathogenic variants to those arising *de novo* when the parents are thought to be unaffected^10^. However, despite impressive analysis of large rare disease cohorts^8,11–15^, genetic diagnostic yield is relatively low. The low hanging fruit, genetic disorders common enough to aggregate in these cohorts, have largely already been discovered, leaving those too rare to find by enrichment alone. There is a need for alternative strategies to identify causal variants directly from a patient’s sequencing data, without relying on frequency of observations in large cohorts. In this work, we present how probabilistic modelling of diverse sequencing efforts provides an answer with the potential to enable clinical interpretation of never-before-seen variants.

Recent work using deep unsupervised models trained only on evolutionary sequences have shown strong promise for accurate clinical variant effect prediction^1–6^ and have demonstrated comparable accuracy to experimental approaches^1^. In addition, since these models do not depend on functional or clinical labeling, they can generalize to variants in genes without previous annotation. However, although these models often perform well in terms of separating *Benign* from *Pathogenic* clinical labels in known disease genes, they are overall not calibrated well across the human proteome. Consequently, previous methods excel at identifying variants that disrupt the function of the resulting protein but do not necessarily predict if it is detrimental at the organismal level.

Variant severity lies on a spectrum: disruption of function in one protein could have modest effects late in life, while the disruption of another protein can be lethal in childhood. Both can be considered “pathogenic” and correctly identified as such by a model, but when attempting to find the genetic cause of a severe disorder when neither variant is in a previously known gene, it is imperative to be able to distinguish between these two scenarios. Current state-of-the-art variant effect prediction models have not been designed with this spectrum of severity in mind.

To help overcome this, we developed popEVE, a model that places variants on a proteome-wide scale of pathogenicity, enabling us to predict if a variant seen in one gene is more detrimental to human health than a variant seen in another. popEVE leverages deep evolutionary data to achieve missense resolution variant effect prediction and shallow variation across the UK Biobank^7^ population to transform the score to reflect human-specific constraint. We identify score thresholds corresponding to moderate and severe pathogenic variants, based on the distribution of scores seen in a meta-cohort of patients with severe developmental disorders, without recourse to clinical annotation. Further analyzing this cohort, we find variant-level evidence for at least 119 novel genetic disorders. 44% more than previously identified in the same cohort, with uncanny functional similarity to known developmental disease genes. By providing a pathogenicity spectrum rather than binary classification, the model presents a new perspective on the distribution of fitness effects in the population and across the proteome.

## Results

### A unified model of population and evolutionary sequences.

**Figure 1.**
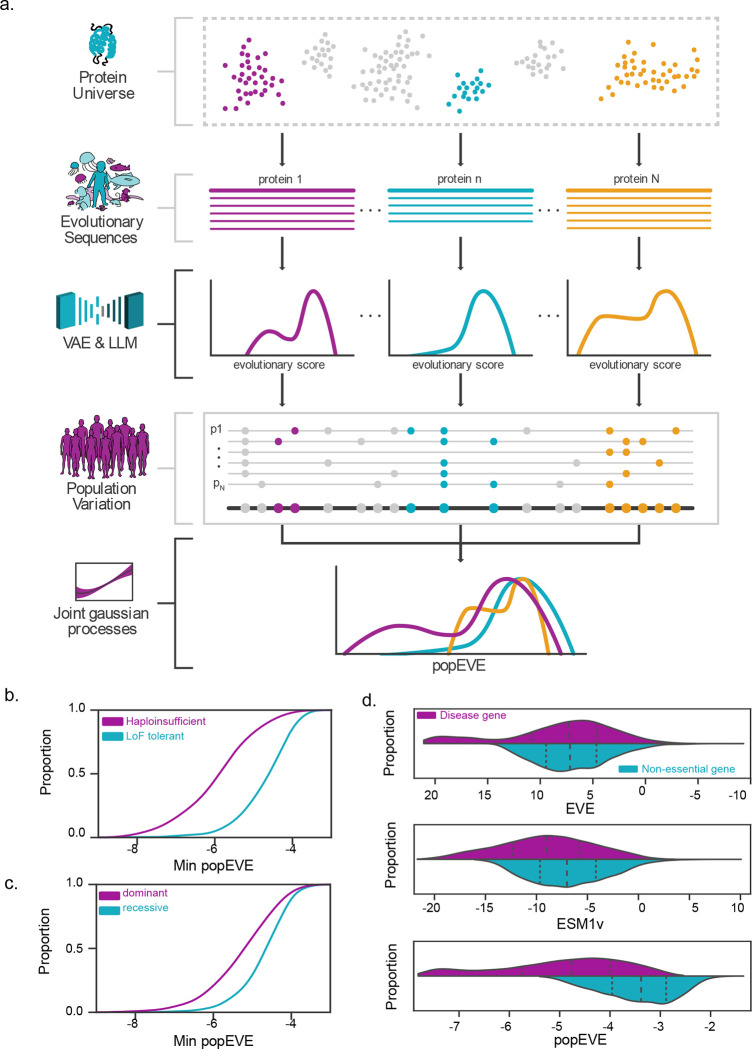
popEVE combines deep evolution and human variation. a. popEVE combines variation from across evolutionary sequences, modeled with EVE and ESM1v, with variation within the human population (UK Biobank), using a joint gaussian process to learn the relationship between evolutionary scores and missense constraint. b. Minimum, i.e. the most deleterious, popEVE score per gene can be used as a measure of gene constraint to distinguish between ClinGen haploinsufficient genes (n=186) and homozygous LoF tolerant genes (n=263) (ks=0.59, p=3e-40) (15,16). c. Minimum popEVE scores for genes with dominant (n=621) and recessive (n=1043) inheritance. Genes with dominant inheritance patterns have more pathogenic scores than genes with recessive inheritance (ks=0.32, p=1e-36) (17,18). d. To illustrate the effect of population adjustment, we compare the distribution of scores in a disease gene, MEF2C, and a non-essential gene, ORF2A14: the bulk of the evolutionary scores overlap for both EVE (top) and ESM1v (middle), with the most deleterious variants in the non-essential gene being equivalent to the moderately deleterious scores in the disease gene. This overlap no longer exists after adjustment (bottom), with the deleterious end of ORF2A14 no longer falling in the likely disease-causing score range.

popEVE is designed to provide a human-specific measure of pathogenicity that enables the effect of variants to be compared across the entire proteome. It leverages both cross-species data and human variation data during training, with the former enabling missense resolution predictions and the latter enabling a proteome-wide human-calibrated measure of pathogenicity (Fig. 1a, Supp. Fig. 1 & 2).

To achieve missense resolution predictions, popEVE incorporates two classes of models that learn from the observed universe of protein sequences: alignment-based models, EVE^1^, and large language models, ESM1v^16^. Despite comparable performance on numerous tasks (Supp. Fig. 3, 4), variant scores from these two types of models are not highly correlated, indicating that they learn different properties and constraints from the same type of data. As such, combining the two should improve variant effect prediction.

To achieve a score which reflects the constraint on missense variants in humans, we train a model to predict the presence of a variant in the UK Biobank, conditioned on the scores from EVE or ESM1v. We model the functional dependence of observing a variant on the underlying EVE/ESM1v score using a latent Gaussian process prior. The inferred non-linear function maps the scores from EVE and ESM1v onto a new scale which, by virtue of training on whole exome data, means that variant scores are now calibrated across proteins to reflect the degree of human specific constraint acting on that variant. Additionally, we employ a zero mean-function to minimize the risk of over-predicting pathogenicity. This pushes variant scores towards neutrality unless there is sufficient evidence to infer otherwise.

The model has two properties which are designed to minimize sensitivity to population structure. First, we model presence of variants in the UK biobank, as opposed to frequency information. Second, the latent Gaussian process is solely a function of the score from EVE/ESM1v and hence lacks the freedom to adjust the score for a single variant in isolation.

The contrast between the popEVE score distributions of the well-known oncogene MEF2C and the non-essential olfactory receptor ORF2A14, illustrates the benefit of population adjustment (Fig. 1d). With EVE alone, the scores of ORF2A14 and MEF2C overlap at the deleterious end of the distribution, which is unexpected due to their strikingly different phenotypic effects in humans. After incorporating population data, the bottom 50% of substitutions no longer overlap. popEVE, in particular the most pathogenic popEVE score per gene, captures disease gene characteristics. This popEVE summary metric significantly distinguishes between ClinGen haploinsufficient genes^17^ and homozygous loss-of-function-tolerant genes^18^, and, genes with autosomal dominant or autosomal recessive inheritance patterns^19,20^ (Fig. 1b & c, Supp. Table 1).

Finally, we note that popEVE preserves, or improves on, its underlying EVE and ESM1v models at protein level tasks, including distinguishing Benign and Pathogenic labels from ClinVar, and correlation with deep mutational functional scans (Supp. Fig. 3 & 4, Supp. Table 2). These tasks are only mildly affected by the score transformation inferred from population data but are sensitive to the benefits of ensembling EVE and ESM1v. Thus, while the primary objective of this method development relates to proteome-wide prediction (next section), we conclude that popEVE achieves a new state-of-the-art performance at these tasks as well.

### Low popEVE scores are enriched in developmental disorders.

**Figure 2.**
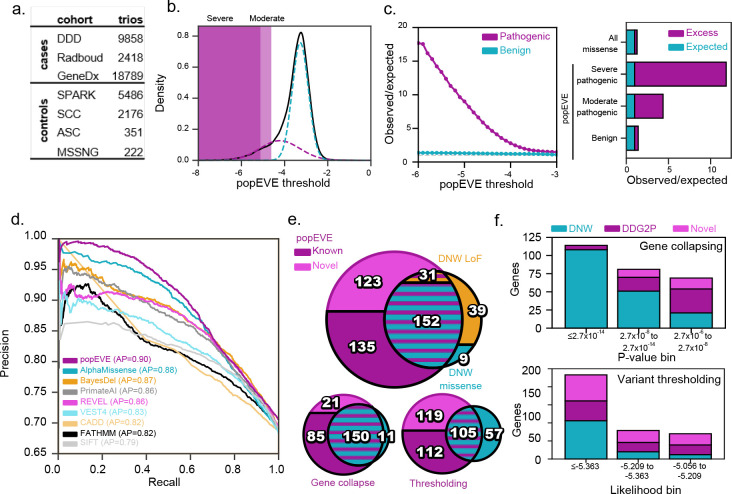
123 new popEVE genes identified in severe developmental disorder cohorts. a. Number of individuals in the severe developmental disorder (SDD) metacohort(6) and controls unaffected siblings from autism spectrum disorder cohorts(20). b. The distribution of popEVE scores for DNMs in SDD cases and unaffected controls (black) is skewed towards pathogenic scores while the bulk are in the benign range. A two-component gaussian mixture model is used to calculate a threshold of severe pathogenicity at 99.99% likelihood of being in the pathogenic distribution at −5.056 and a moderate threshold at 99% likelihood at −4.617. See Supp. Fig. 5a for distributions for cases and controls separately. c. With increasingly severe thresholds, pathogenic DNMs in the SDD metacohort are significantly enriched versus expected given the background mutation rate (left). At our severe threshold, popEVE pathogenic variants exhibit over 15-fold enrichment while popEVE benign variants are in line with expectation (right). Moderately pathogenic variants are enriched 5-fold. d. popEVE is better at separating SDD cases with at least one missense variant in a known DD gene from controls than other predictors with an average precision of 88%. e. Both popEVE gene- and variant-association methods achieve an 80% recall when compared to the DeNovoWEST (DNW) analysis of the same cohort and a 95% recall of the DNW genes discoverable solely with missense variation. There is a greater overlap between popEVE gene-collapsing and DNW than the thresholding approach, as gene-based methods discoveries had a minimum of three DNMs while thresholding returns 67 and 46 genes with variants present solely in a one or two patients respectively f. More genes are previously discovered in the SDD by DNW or elsewhere in the DDG2P at higher levels of significance, either by p-value in gene collapsing (top) or likelihood of pathogenicity for variant thresholding (bottom).

We applied popEVE to a set of missense de novo mutations (DNMs) from 31k individuals with severe developmental disorders, a metacohort combining trios from the Deciphering Developmental Disorders Study^12^, GeneDx, and the Radboud Medical Center^8^, to prioritize causal variants and discover novel disease gene candidates (Fig. 2a). For further comparison, we include a set of controls – unaffected siblings from four separate autism spectrum disorder cohorts^9^. After reannotation with VEP^21^, cases and controls and 19,821 and 3,045 unique missense variants covered by popEVE (Supp. Table 3).

To establish a score threshold for classifying DNMs as candidates for causing disease, we fit a two-component Gaussian mixture model to the distribution of popEVE scores across all cases and controls without using their labels (Fig. 2b and Methods). We selected a high confidence threshold (−5.056) where 99.99% of the variant scores are in the low fitness distribution. These 1163 variants are over 15-fold enriched in the SDD cohort versus the expected number of pathogenic DNMs given the background mutation rate (Fig. 2c), a five times higher fold enrichment than other state-of-art methods, e.g. primateAI-3D at 2-fold^22^. Furthermore, even variants we define as moderately pathogenic are five times enriched in the developmental disorder cohorts, again outperforming previous methods. popEVE also differentiates cases from controls over a range of recall fraction (average precision 0.88), better than any other state-of-the-art variant effect predictors, even including those methods that train on clinical data or minimally use the clinical data to draw thresholds (Fig. 2d, Supp. Fig. 5 b, Supp. Table 2). Taken together this initial analysis of popEVE of the three developmental disease cohorts analyzed here demonstrates state-ofthe-art performance without risking lack of generalizability.

### 119 novel candidate genes at variant resolution.

We used a two-pronged approach to discover associations: (1) thresholding the scores for more than a 99.99% likelihood of falling within the low fitness distribution (2) gene collapsing, comparing variant scores seen in the cohort to what is expected given the background mutation rate and the spectrum of popEVE scores within and across proteins (Methods).

This results in 442 genes including 183 that were identified by previous study of same cohort^8^ (Fig. 2e&f, Supp. Table 4). popEVE recalls 94% of genes previously identified in the three cohorts and over half (135) of the novel discovery gene set have been associated with developmental disorders from other studies in the Development Disorder Genotype - Phenotype Database^23^ and the distribution of scores of the variants in the remaining 124 novel genes is similar to the distribution of those from known genes. Interestingly, we recover 31 genes using missense variants alone where previous work only identified the gene for patients with loss of function annotation. Of the 50 previously known genes that we missed by variant alone but recalled with gene collapsing, many have moderately pathogenic scores. which motivates using the two-pronged approach. Taken together we provide evidence that variant score thresholding alone can provide accurate results but that the modeling allows both methods to be used in practice.

Through score thresholding at 99.99% probablity of being in the more deleterious distribution, we identified variants in 119 novel genes (Supp. Table 5) associated with severe developmental disorders in these cohorts and explored whether there is independent functional and structural evidence for our results. First, we looked at the functional convergence of our discoveries with known developmental disease genes. Most of the top significant functional annotations for the novel genes that are non-trivial are shared with known genes (using DAVID^24^, Supp. Table 6 & 7). These include annotations such as chromatin organization (n=15), DNA binding and ATP binding (n=29), metal ion binding (n=32), and histone binding (n=7).

**Figure 3.**
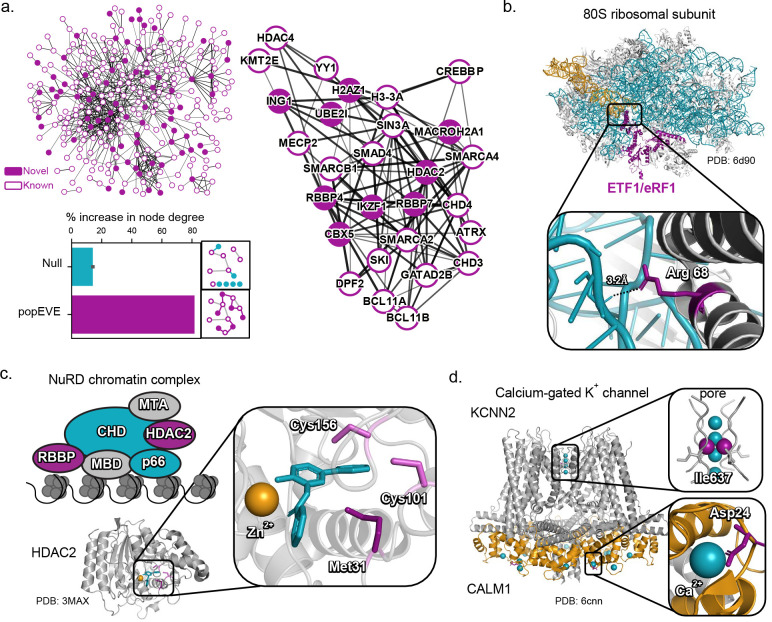
Structure and function analysis supports accuracy of new discoveries. a. Novel genes discovered with popEVE (by variant thresholding alone) have many known biochemical interactions (defined using STRING(25)) with genes previously associated with disease from the three cohorts(6), forming a dense network of previously known and newly discovered genes, see also Supp. Fig. 6 (left top), including genes involved in chromatin modeling complexes (middle) and with an 80% increase in node degree as compared to random sets of the same number of genes which saw an average of 14% (with p=0, t-test (left bottom)) see also Supp. Table 8. The densest portion of the subnetwork includes many of the genes involved in the NuRD complex (right) b. The top scoring variants Arg68Leu and Arg192Cys in ETF1/ erF1( a translation termination factor) are contacting the anticodon site and the peptidyl transferase site in the ribosomal RNA, ternary complex (PDB:6d90(27) c. the novel discovery HDAC2 interacts with many genes known in severe developmental disorders including those in the NuRD complex where the variant Met31Arg is proximal to the foot-pocket of the active site (PDB:3max(26)) d. novel discoveries KCNN2 and CALM1 both contain highs scoring variants in functional sites - Ile637Phe in the highly conserved T(V/I)GYG pore motif and Asp24Tyr in CALM1 which chelates the Ca^2+^ in the wild type (homologous complex structure PDB: 6cnn(28)) see Table 1.

Since the functional overlap was so striking, we then asked whether the new genes interact directly with those previously known. We found evidence that for 67 of the 130 new discoveries have a known direct interaction with the 285 previously identified genes based on experimental data^25^. Taking the whole interaction network, the density of interactions doubles compared to adding random sets of genes when the newly discovered set is added (1.6 on average to 3.1 p=0 for direct interactions; Fig. 3, Sup Fig. 7, Supp. Table 8). An example of this increased density is the chromatin-associated complex, NuRD – crucial in embryonic development. This complex contains several genes associated with developmental disorders, including CHD3/4 and MTA. We discover four new genes in NuRD complex including HDAC2, RBBP4, RBBP7 and IKZF1 and five in another chromatin associated complex, Sin3 complex (Supp. Table 8). HDAC2 itself interacts with 8 previously known developmental disorder-associated genes and it’s top scoring variant, M31R, lies in the ‘foot pocket’ of the acetylase active site^26^.

If the mutated residues are important for the function of the protein, we should be able to analyze this at the atomic level i.e., in 3D structures in the context of its interactions. Comprehensive structural analysis revealed that 87% of these new discoveries are within 8Å of an interacting protein, metal, ligand or cofactor, Table 1 and Supp. Table 9 (100/116 of the newly discovered genes have a high confidence 3D structure in a complex with a ligand).

For example, the amine of R192 and R68 in ETF1 (eRF1) are close (3.2Å) to the phosphate backbone of RNA in the eRF1-eRF3-GTP ternary complex that mediates translation termination (6d90^27^; In the calcium gated potassium ion channel, KCNN2 (modeled on KCNN1, PDB: 6cnn^28^). Ile637 is part of the T(V/I)GYG pore motif that is essential for ion transport. Another top variant, D24Y in CALM1 (PDB: 6cnn^28^) has a mutation that is 2.4 angstroms from the activating Ca^2+^. An automated assessment of the novel genes finds that the vast majority (87 out of 100 with resolved structures and interaction partners) have candidate variants in close three-dimensional distance (<8Å) to residues on their interacting partner. Both analyses indicate that it is unlikely that these candidate variants are in functional sites by chance.

Taken together, (1) the recall of genes identified in previous analyses of the same cohorts, (2) the functional overlap with known developmental genes and (3) the structural evidence supports the popEVE discovery variants and genes.

### popEVE predicts a sparse distribution of severe pathogenic variants.

Approaches to clinical variant annotation to date place great emphasis on segregating variants as either (Likely) Benign or (Likely) Pathogenic. While this “binary” approach to variant interpretation has some benefit for clinical decision making, it obscures the fact that some variants will lead to more severe disease phenotypes than others. popEVE predicts a spectrum of pathogenicity, reflecting variant level constraint in the human population, and thus presents a novel perspective of the distribution of disease-causing variants across the proteome. Based on these predictions, we explored the distribution of predicted severe and moderately pathogenic variants across the proteome, across UK biobank participants, as well as the relation of this score to known disease causing variants and notions of severity such as age of onset and death.

**Figure 4.**
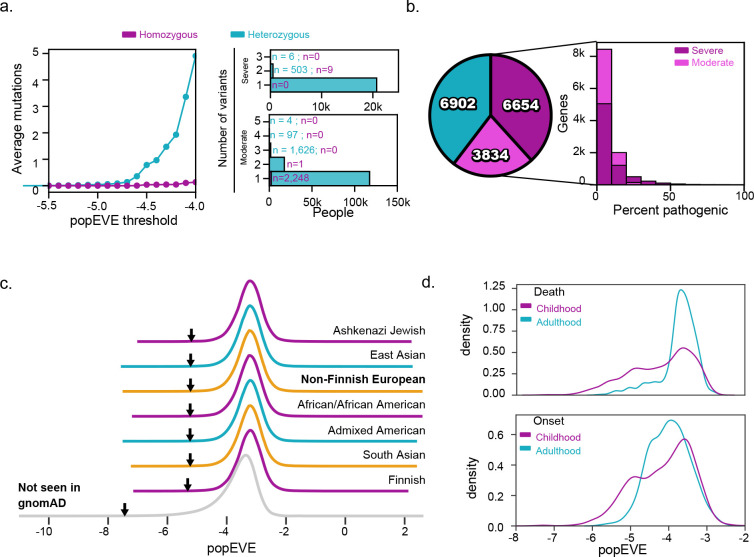
popEVE predicts a broad spectrum of disease severity. Predictions are robust and applicable across populations. a. In the UKbiobank, individuals have at most one homozygous and up to three heterozygous severely pathogenic variants – 96% of the 500k individuals have no severely pathogenic missense variants (left). About 72% of UKBB individuals have no severely or moderately pathogenic variants and at most five moderately pathogenic variants. On average, individuals have 0.222 and 0.434 severely and moderately pathogenic variants respectively. As the score moves past our moderately pathogenic threshold of −4.617, the average number of variants per person plummets to below 1 on average per person. b. A little over half of autosomal genes harbor at least one severely pathogenic variant or at least one moderately pathogenic variant (left). Only a small number of all possible mutations in these genes are pathogenic – on average 5% and 6% severely or moderately pathogenic respectively (right). c. The distribution of popEVE scores for rare variants (AF<0.01) is consistent across populations found in gnomAD, indicating that despite using primarily non-Finnish European subjects for score adjustment there is no population bias. Variants not seen any gnomAD population are in grey. The 99.9% percentile for each distribution is marked with an arrow. d. ClinVar pathogenic variants (with at least 1 star curation rating) in phenotypes associated with early onset and premature death in childhood have more deleterious popEVE scores than those associated with later onset and death after maturation. There is a greater separation between the distribution of variant scores for age of death (ks=0.34, pvalue=7e-41) than onset (ks=0.19, pvalue=2e-10). Onset and death labels were acquired from OrphaNet

popEVE predicts 39% of genes have at least one high-confidence pathogenic missense variant; a little over a quarter of these genes have known disease associations according to OMIM^29^ or DDG2P, indicating there are many disease-gene associations yet to be discovered. However, these variants are sparse, constituting only 3% of possible missense variants (Fig. 4a). 4% are predicted moderately pathogenic, with the remainder predicted to be ambiguous or benign. This sparsity of missense pathogenicity may make methods such as burden testing challenging as they are likely to be underpowered even in large cohorts.

Only 4% of UK Biobank participants have at least one high-confidence pathogenic variant (or 0.04 per person) while 31% (0.31 per person) have at least a moderate or higher-confidence variant (Fig. 4b). For comparison, observed participants of the Regeneron Genetics Center Million Exome dataset^30^ carry an average of 1.6 high-confidence ClinVar pathogenic variants. This suggests that our definition of pathogenicity is more heavily selected against in the population than ClinVar Pathogenic variants. The average number of variants per person plummets around our moderately pathogenic threshold, indicating that the threshold is generalizable across cohorts.

Variant effect predictors must be able to distinguish benign but recent variants from those that are rare due to purification of deleterious mutations. Distributions of popEVE scores for rare variants are strikingly similar across gnomAD^31^ populations (Fig. 4c), suggesting popEVE is robust to population structure, with no indication that training on predominantly European cohort imparted bias in the variant scores.

To further investigate the spectrum of severity, we compared the distribution of popEVE scores for ClinVar pathogenic variants in genes which have also been associated with early onset or premature death in childhood to diseases, with later onset or death during adulthood^32^. The distribution of scores of ClinVar pathogenic variants seen in genes associated with these early onset/death phenotypes show a significant shift in the deleterious-end of popEVE scores (Fig. 4d). However, the majority of variants fall within the ambiguous/benign range of popEVE, likely due to differences in inheritance, penetrance and severity, and, as expected, these variants are primarily involved in diseases with recessive inheritance patterns (Supp. Fig. 7). This difference is particularly striking for premature death, with dominant diseases significantly separating from recessive diseases no matter the age of death.

### popEVE pinpoints candidate variants without trios.

As popEVE does not overestimate pathogenicity of germline variants in the population at large, we then asked whether our results can be computed directly from the child genome alone without parental genetic information. To test our model’s ability to identify causal mutations without de novo labels, we investigated rare variants (MAF < 0.01), both inherited and de novo, from a subset of the severe developmental disorder meta-cohort, almost 10k individuals from the Deciphering Developmental Disorders study^12^. According to our model, 12% of this cohort has at least one severe pathogenic variant, three-fold more than in the 4% in the general population. On average the popEVE pathogenic DNMs are more deleterious than the pathogenic inherited mutations (Supp. Fig. 8). For 98% of the 513 individuals with a severe de novo missense, the DNM ranks the most pathogenic, while for the remainder it is the second most pathogenic – thus, without knowing their de novo status, the candidate DNMs would still rise to the top of the variant ranks. If we take the most pathogenic variant per patient, we still recover 95% of the variants and genes identified by variant thresholding of the *de novo* variants alone, demonstrating popEVE’s power to identify the likely most deleterious mutation. As for the genes with severe pathogenic inherited variants, 38% of the 402 are already known to be associated with developmental disorder and 29 are among the 124 novel genes discovered in the full meta-cohort. Even if the inherited variants in these genes are less likely to be causal as most parents are unaffected, they may contribute to the observed phenotypes in more complex genotypes, suggesting further investigation.

## Discussion

Patient sequencing has become standard for many diseases in several countries, with growing accessibility worldwide. Hence, there is an urgent need for variant interpretation strategies that are broadly applicable and can provide guidance even in cases where just one individual is suspected to have the disease. Studies seeking to enroll individuals with rare diseases allow them to become aggregate in common, and, as such, standard methods of genetic burden and enrichment become viable for discovering novel gene-disease associations. However, there remains a long tail of cases so rare they may be unique. In this work we developed a model to aid in the genetic diagnosis of these patients.

In recent years, there has been a surge of models capable of predicting whether variants are benign or pathogenic. However, in this area, consideration for the heterogeneity of severity and penetrance of disease-causing variants has been largely absent. Here, we explored the possibility that, in some situations, it may be beneficial to consider variants as lying on a spectrum of pathogenicity. To capture this spectrum, a model must be capable of ranking variants both within and across distinct genes, i.e., a model of the whole proteome. While several models provide proteome-scale predictions, to our knowledge, popEVE is the first to be built specifically to calibrate scores to be comparable across genes, and hence, may be regarded as the first, albeit simple, model of the human proteome.

To advance whole proteome modeling, there is a long road of necessary future developments. The next, perhaps most obvious, step is to account for protein-protein interactions, analogous to the development of protein-level models. Where early models considered each position in the sequence as statistically independent (e.g. column conservation models using MSAs), only later did models account for epistatic and higher-order interactions. Another clear limitation of this model as a representation of the whole proteome is the inability to assess loss of function variants, such as nonsense or truncation mutations, and, thus, are unable to compare their severity to missense variation. To the best of our knowledge, no unified model of loss of function and missense variants with sufficient predictive power currently exists. We note, however, that due to the modular nature of the model, it would be straightforward to incorporate such a model, should one become available in the future. In other words, the human proteome-calibration underlying popEVE is independent of the form of genetic variation and can easily be expanded.

Despite the simplicity of popEVE, it presents multiple opportunities for diagnosis and studying the genetic underpinnings of disease more broadly. When applied to the severe developmental disorder metacohort, we found evidence of 104 genetic disorders that burden testing was underpowered to detect. Through complementary gene enrichment and network analysis, we find many of these genes are intimately related to genes whose role in developmental disorders is already established, providing further evidence that these genes do indeed indicate novel genetic disorders. More broadly, the model predicts that a large number of genes are capable of giving rise to severe phenotypes, implying that there are still many genetic disorders yet to be identified or even seen. A similar conclusion is reached in Kaplanis et al^8^ but via a distinct analysis. Here we clarify this forecast by predicting which genes and variants are most likely to be involved.

Finally, we must note the detrimental impact of building large-scale proteome or genome models; we are reaching a point where energy and computational consumption of developing and training models is costly both financially and environmentally^33^. In this work, we sought to use a modular approach, enabling us to repurpose previous models, as well as easily update components of the model with future developments at a minimal computational cost. Deep learning strategies with these properties are currently scarce, and we urgently need more techniques that lend themselves to reducing computational costs or have components that can be readily reused or recycled.

## Figures and Tables

**Table 1.** Top 20 most pathogenic variants in novel discoveries with resolved structures.

gene	mutant	score	PDB ID	interacting partner	distance (Å)

ETF1	R192C, R68L	−7.2, −6.8	6d90	18s ribosome	1.6, 2.7
RBBP4	H373R, T155I	−6.8, −6.0	6zrd, 2xu7	MTA1, ZFPM1	3.7, 2.9
WDR5	S62N	−6.8	7u9y	benzamidine	6.7
UBE2D3	S105Y	−6.7	6t7f	ubiquitin	2.1
EIF4A2	Q60K	−6.6	3ews	ATP	1.9
UBE2H	D120V	−6.2	6zhs	E1	2
XPO1	T448K	−6.2	3nc1	RAN	2.9
COPS2	F69C	−6.1	6a73	inositol hexakisphosphate	6.5
RBBP7	N325D	−6	6g16	MTA1	3.3
DDX17	V484M	−5.9	6qdv	ATP	2.6
SPIN1	Y170C	−5.9	7bqz	H3.2	3
WARS1	G163V	−5.8	1r6t	Trp-AMP	2.7
MAT2A	S206F	−5.9	4ktt	SAM	2.4
KCNN2	I637F	−5.8	6cnn	K^+^	2.7
ZMYND8	R333G	−5.8	4cos	Zn^++^, debrin	5.5
ACTC1	S340F	−5.8	7tj7	MYBPC3	4.7
PSMA2	G125D	−5.8	8cvt	PSMA4	2.9
MAP2K4	S262N	−5.8	7m0y	trametinib	2.9

## Data Availability

Interactive web viewer and downloads for popEVE scores available at pop.evemodel.org. Code will be available at github.com/debbiemarkslab/popEVE.
